
JOURNAL INFORMATION
==============================
NLM Title Abbreviation: Behav Neurol
Journal ID: Behav Neurol
NLM Journal ID: 8914585
Journal ID: BN

Behavioural Neurology

ISSN: 0953-4180
EISSN: 1875-8584
Publisher: Wiley

ARTICLE INFORMATION
==============================
PMCID: PMC5434360
PMID: 21422559
DOI: 10.3233/BEN-2010-0299
Article ID: 925161
Article version: 1
Subjects: Research Article

Induction of Semantic Impairments Using rTMS: Evidence for the Hub-And-Spoke Semantic Theory

Pobric Gorana 1 *
Jefferies Elizabeth 2
Ralph Matthew A. Lambon 1
1 Neuroscience and Aphasia Research Unit School of Psychological Sciences University of Manchester Manchester UK
2 Department of Psychology University of York and York Neuroimaging Centre York UK
*Gorana Pobric: gorana.pobric@manchester.ac.uk
Print publication date: 2010
Electronic publication date: 2011 Mar 7
Volume: 23
Issue: 4
First page: 217
Last page: 219
Received 2011 Jan 31; Accepted 2011 Jan 31
Copyright: Copyright © 2010 Hindawi Publishing Corporation and the authors.
Copyright year: 2010
License: This is an open access article distributed under the Creative Commons Attribution License, which permits unrestricted use, distribution, and reproduction in any medium, provided the original work is properly cited.
License URL: https://creativecommons.org/licenses/by/3.0/

==============================
Behavioural Neurology 23 (2010) 217–219
DOI 10.3233/BEN-2010-0299
IOS Press

### Induction of semantic impairments using rTMS: Evidence for the hub-and-spoke semantic theory

Gorana Pobric[a,*], Elizabeth Jefferies[b] and Matthew A. Lambon Ralph[a]
[a]*Neuroscience and Aphasia Research Unit, School of Psychological Sciences, University of Manchester, Manchester, UK*
[b]*Department of Psychology, University of York and York Neuroimaging Centre, York, UK*

### 1. Introduction

Semantic cognition permits us to bring meaning to our verbal and nonverbal experiences and to generate context-and time-appropriate behaviour [1,2]. It is core to language and nonverbal skilled behaviours (e.g., using objects), and when impaired after brain damage, it generates significant disability [3]. A fundamental neuroscience question, therefore, is how does the brain code and generate semantic cognition? Historical and some contemporary theories emphasise that conceptualisation stems from the joint-action of modality-specific association cortices (the "distributed" theory) [4,5] reflecting our accumulated verbal, motor and sensory experiences. Parallel studies of semantic dementia, rTMS in normal participants and neuroimaging indicate that the anterior temporal lobe (ATL) plays a crucial and necessary role in conceptualisation by merging experience into an amodal semantic representation [1,2,6–8].

To date, studies have focussed on the contribution of either modality-specific association regions or the ATL to semantic memory. In this rTMS investigation, we tested the differential contribution of both regions for the first time. Like neuropsychological studies, rTMS can be used to test the necessity of regions for cognitive function. It has the additional, unique benefit that the functional contribution of multiple regions can be successively compared within the same participants. We used this approach to delineate between three different hypotheses: (a) *distributed-only* – concepts reflect the conjoint action of modality-specific areas alone without the ATL; (b) *hub-only* – in which concepts are formed within the ATL and modality-specific regions only provide sensorimotor input/output gateways rather than making a necessary contribution to conceptualisation; (c) *hub-and-spoke* – in which modality-specific regions provide the basic sensory, motor and verbal ingredients whilst the ATL hub supports an additional amodal representation which codes the pan-modal, deep statistical structure and thus generates a high-dimensional, modality-independent similarity matrix [1,8]. Under this latter account, both the ATL "hub" and modality-specific "spokes" provide necessary and important contributions to conceptualisation.

We adjudicated between these competing theories by investigating category-specific impairments. To date, these have only ever been observed clinically; some patients present with relatively greater problems for one domain than another (e.g., poorer performance for animals than manmade items) [9,10]. The feature-weighting hypothesis [9] proposes that categories are reliant on different sources of information, coded by modality-specific regions. This provides a method for testing the different theories of conceptualisation. Specifically, we compared the effect of stimulating the

*Corresponding author: Dr. Gorana Pobric, Neuroscience and Aphasia Research Unit (NARU), School of Psychological Sciences (Zochonis Building), University of Manchester, Oxford Road, Manchester, M13 9PL UK. Tel.: +44 161 275 1967; Fax: +44 161 275 2873; E-mail: gorana.pobric@manchester.ac.uk.

ISSN 0953-4180/10/$27.50 © 2010 – IOS Press and the authors. All rights reserved

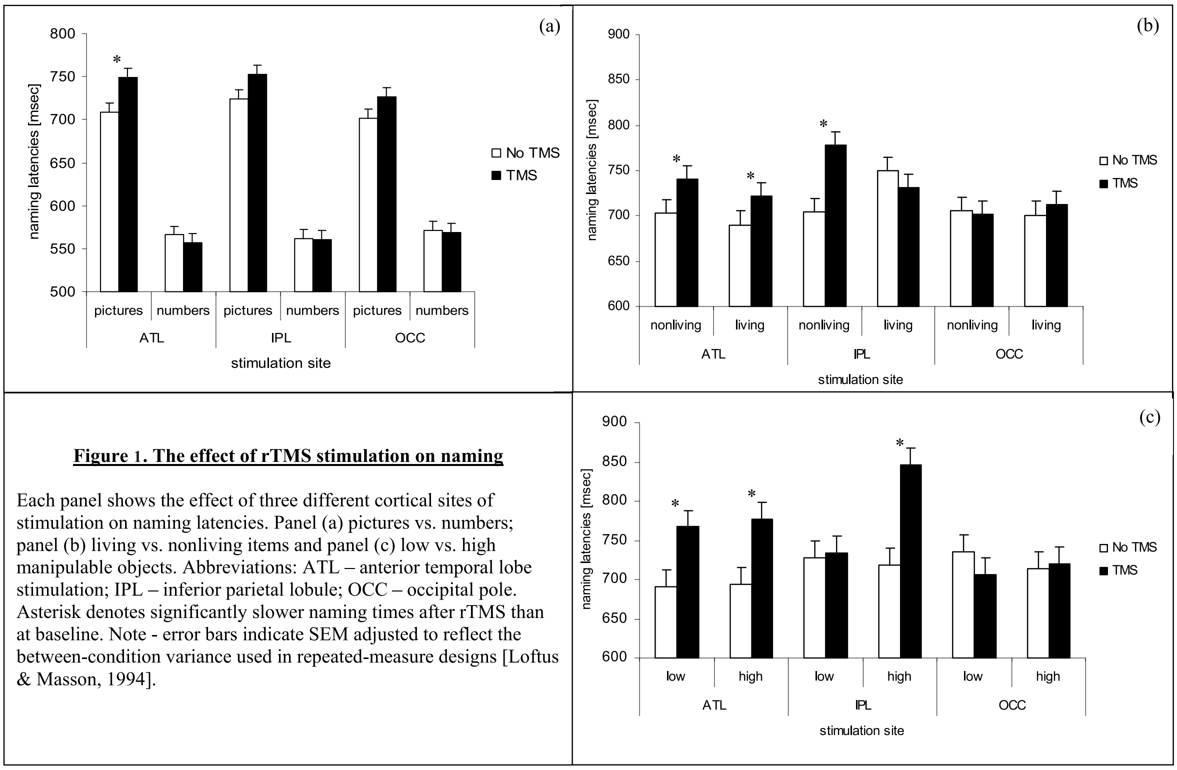

Fig. 1. The effect of rTMS stimulation on naming.

ATL and IPL in normal participants (by applying rTMS off-line for 10 min at 1-Hz (600 s at 120% motor threshold level) over left ATL ($-53$, 4, $-32$), left IPL ($-49$, $-44$, 48) and occipital pole (Oz) prior to naming pictures and number. If the ATL is involved in semantic memory, as proposed, then rTMS should generate a category-general effect. If the IPL spoke is implicated then stimulation should impact on semantic performance but only for concepts that rely on praxis information – i.e., manipulable manmade objects. Thus stimulation at this site should induce a category-specific impairment which, as far as we are aware, has never been demonstrated before in neurologically-intact participants.

ATL stimulation generated a selective slowing of basic level naming [t(8) $= -3.3$, $p < 0.05$] but had no impact on number naming, adding to previous evidence for the involvement of the ATL in semantic processing [6]. Stimulation of the occipital control site had no impact on either task, indicating no generalised, non-specific effect of rTMS (see Fig. 1a).

To examine emerging category effects from original 200 items we created two lists of living and non-living items matched for familiarity, frequency and visual complexity. Each stimulation site produced significantly different effects on the two categories. There was a category-general effect for ATL stimulation [naming times slowed for living t(8) $= 2.391$, $p < 0.05$] and nonliving items [t(8) $= 2.394$, $p < 0.05$]. In contrast, left IPL stimulation only slowed responses for nonliving items [t(8) $= 3.1$, $p < 0.05$]. Again there was no effect after rTMS to the occipital control site (see Fig. 1b).

To confirm that the IPL category effect reflected the coding of praxis information, we compared the effect of rTMS to two matched sets of manipulable vs. non-manipulable manmade items. The same pattern emerged across the three sites: ATL stimulation slowed both sets of manmade items [low manipulable (t(8) $= 2.34$, $p < 0.05$) and high manipulable items (t(8) $= 2.31$, $p < 0.05$)]. Crucially, left IPL stimulation slowed responses for high manipulable items only [t(8) $= -4.21$, $p < 0.05$]. In addition, TMS delivered to the occipital control site had no significant effects on naming times (see Fig. 1c).

In summary, through the use of rTMS in normal participants, we have been able to demonstrate two contrasting effects. Stimulation of the ATL leads to a generalised slowing of semantic processing across all types of concept (living, manipulable objects and non-manipulable items). This is in keeping with the category-general effects observed in semantic dementia [1–3]. In contrast, stimulation to the IPL generates a category-specific pattern reflective of the praxis information coded in this neural region. As far as we are aware, this is the first time that a category-specific deficit has been generated in normal participants.

In conclusion, the findings of this study fit squarely with the *hub-and-spoke* model of semantic memory and rule out the other models. Both the ATL amodal hub and the modality-specific association "spokes" contribute to semantic representations. Because the ATL hub is involved in the translation and deeper encoding of pan-modal information sources, the representations become modality-invariant [1,2,9] and thus they are involved in conceptualisation for all types of category. In contrast, modality-specific sources of information only contribute to the subset of concepts that are experienced in that modality. Unlike of modality-specific areas, IPL is an ideal test-bed for this given that there is an almost binary division of praxis experience between manipulable objects and other concepts.

### Acknowledgements

The study was supported by an RCUK fellowship, a Wellcome project grant (078734/Z/05/Z) and an MRC programme grant (G0501632).

### References

[1] M.A. Lambon Ralph and K. Patterson, *Ann NY Acad Sci* **1124** (2008), 61–76.
[2] K. Patterson, P.J. Nestor and T.T. Rogers, *Nat Rev Neurosci* **8** (2007), 976–987.
[3] J.R. Hodges, K. Patterson, S. Oxbury and E. Funnell, *Brain* **115** (1992), 1783–1806.
[4] G.H. Eggert, *Mouton*, The Hague (1977).
[5] A. Martin, *Annu Rev Psychol* **58** (2007), 25–45.
[6] G.G. Pobric, E. Jefferies and M.A. Lambon Ralph, *PNAS* **104** (2007), 20137–20141.
[7] J.R. Binder, R.H. Desai, W.W. Graves and L.L. Conant, *Cereb Cortex*, (2009).
[8] T.T. Rogers et al. *Psychol Rev* **111** (2004), 205–235.
[9] E.K. Warrington and T. Shallice, *Brain* **107** (1984), 829–854.
[10] B.Z. Mahon and A. Caramazza, *Annu Rev Psychol* **60** (2009), 27–51.
